# Development and evaluation of culturally sensitive psychosocial interventions for under-served people in primary care

**DOI:** 10.1186/s12888-014-0217-8

**Published:** 2014-08-01

**Authors:** Karina Lovell, Jonathan Lamb, Linda Gask, Pete Bower, Waquas Waheed, Carolyn Chew-Graham, Jon Lamb, Saadia Aseem, Susan Beatty, Heather Burroughs, Pam Clarke, Anna Dowrick, Suzanne Edwards, Mark Gabbay, Mari Lloyd-Williams, Chris Dowrick

**Affiliations:** School of Nursing Midwifery and Social Work, University Place, University of Manchester, Oxford Road, Manchester, M13 9PL UK; NIHR School for Primary Care Research, Manchester Academic Health Science Centre, University of Manchester, Manchester, M13 9PL UK; Institute of Psychology, Health and Society, University of Liverpool, Whelan Building, Liverpool, L69 3GB UK

## Abstract

**Background:**

Psychological therapy is effective for symptoms of mental distress, but many groups with high levels of mental distress face significant barriers in terms of access to care, as current interventions may not be sensitive to their needs or their understanding of mental health. There is a need to develop forms of psychological therapy that are acceptable to these groups, feasible to deliver in routine settings, and clinically and cost effective.

**Methods:**

We developed a culturally sensitive *wellbeing intervention* with individual, group and sign-posting elements, and tested its feasibility and acceptability for patients from ethnic minorities and older people in an exploratory randomised trial.

**Results:**

We recruited 57 patients (57% of our target) from 4 disadvantaged localities in the NW of England. The results of the exploratory trial suggest that the group receiving the wellbeing interventions improved compared to the group receiving usual care. For elders, the largest effects were on CORE-OM and PHQ-9. For ethnic minority patients, the largest effect was on PHQ-9. Qualitative data suggested that patients found the intervention acceptable, both in terms of content and delivery.

**Conclusions:**

This exploratory trial provides some evidence of the efficacy and acceptability of a wellbeing intervention for older and ethnic minority groups experiencing anxiety and depression, although challenges in recruitment and engagement remain. Evidence from our exploratory study of wellbeing interventions should inform new substantive trial designs.

**Trial registration:**

Current controlled trials ISRCTN68572159

## Background

Mental health problems such as depression and anxiety impose substantial emotional, social and economic burdens on those who experience them, their families and carers, and society as a whole. Clinical trials have demonstrated the effectiveness of a range of interventions and initiatives in improving outcomes for people experiencing these common but disabling problems. However many people with high levels of mental distress are disadvantaged, either because care is not available to them in the right place and time, or because when they do access care, their interaction with care-givers deters help-seeking, or diverts it into forms that do not address their needs. Various interventions [1], collaborative care [2], self-management [3,4], and social and community initiatives [5] have shown preliminary evidence of effectiveness in trials in improving outcomes for people experiencing depression and anxiety who may not readily access timely or appropriately targeted care.

Developing interventions to widen access to mental health care is a policy priority in the UK [6]. As part of a larger funded programme grant [7], detailed reviews of groups known to experience poor access were synthesized to inform the development of a wellbeing intervention (summarised in methods). The reviews [8,9] emphasised both heterogeneity and commonalities in access issues within and across under-represented groups. The emerging evidence-base accordingly emphasises delivery adaptations (practitioner skills, characteristics and flexible patient centred approaches) over cultural adaptation of underlying principles of therapies [10-12]. While evidence-based culturally adapted treatments have been available for some time, US experience suggests they remain ‘largely unused in usual care settings’ [13]. Designing specific interventions for different under-represented groups through a common framework may improve the subsequent uptake in services [14].

Engaging hard-to-reach groups and providing practitioners with the support to address wider systemic barriers required adjunct primary care and community engagement interventions [7]. These community [15] and systems [16] interventions, engaged the public, NGOs and health professionals in developing and disseminating materials addressing health literacy and cultural competence and provided information and feedback to allow for tailoring and publicising the psychosocial interventions. Success in improving access to care is nevertheless predicated on improving the availability of acceptable and effective therapies.

Ethnic minority groups and older people were selected from the eight exemplar groups included in the review phase for development of and testing of the intervention model in phase two. As part of the design for evaluation of the wider programme, the psychosocial intervention (reported here) and enhanced primary care engagement was active in four different localities, with an active community engagement component in just two [7]. The localities determined the focus, with South Asian communities predominating in one locality, and Somali communities in the other, the two older peoples sites were predominantly White British. All localities were socio-economically deprived areas in the North West of England. Sampling was inclusive, thus Elders sites could recruit both white British and ethnic minority elders to the elders intervention, and ethnic minority sites may recruit elders to the ethnic minority intervention.

Women of South Asian family origin in the UK have a high prevalence of depression and self-harm, often in the context of severe and persistent social difficulties, which only become apparent when they are in a crisis [17]. Depression is common in older people, particularly those with chronic physical illness, but tends to be under-diagnosed and inadequately managed [18,19].

While there are many differences commonalities include the impact of chronicity, social isolation, stigma and a lack of identification with routine biomedical understandings of mental health [20]. A marked difference between groups included meeting the language needs of some minority patients, however deep rooted communication issues such as differential mental health models [21], perceived candidacy for treatment [22] and recursivity in cultural expectations of treatment [23] exist in both groups [8].

### The programme had two distinct phases

Phase 1 aimed to:

- Determine the content and delivery method, of an intervention protocol targeting older and ethnic minority people
- Develop and deliver a training package for mental health workers to effectively deliver the intervention protocol.

Phase 2 aimed to:

- Test the intervention protocol by estimating key parameters for a definitive trial including:

- ○ referral and recruitment rates
- ○ uptake and delivery of the intervention
- ○ outcomes in patients receiving the intervention compared to those in usual care
- ○ acceptability of the intervention from user and provider perspectives

Our aim was to develop and evaluate an, acceptable and culturally sensitive psychosocial intervention for older people, and people from ethnic minority communities. While the samples involved are relatively small they support the feasibility of developing psychosocial interventions to improve access for hard to reach groups driven by a set of common theoretical underpinnings and common mechanisms and practices to support tailoring of interventions to the particularities of local communities.

## Methods

### Phase 1: Developing the intervention

**Figure 1:**
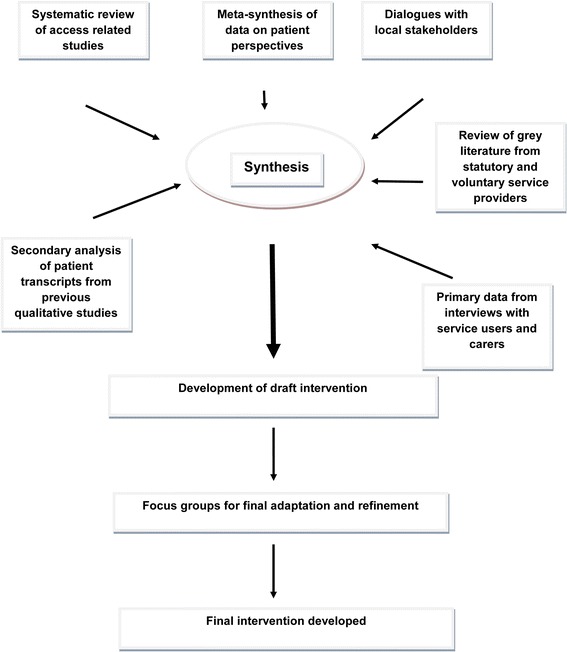
**Developing the wellbeing intervention.**

We summarise our methods of developing the intervention in Figure 1. We drew on six sources of evidence: a systematic review of access studies, a meta-synthesis of data on patient perspectives, dialogues with local stakeholders, a review of grey literature from statutory and voluntary service providers, secondary analysis of patient transcripts from previous qualitative studies [21,24] and interviews with service users and carers [9,20]. The research team held a one-day workshop to synthesise the key findings of the 6 sources of evidence. We drew up a matrix of results, with each row of the matrix detailing one of the key intervention design issues that we wished to address, and the columns referring to the results from each individual data set [8]. This matrix was used to derive the key principles to be incorporated into the intervention. These included:

- Evidence-based psychosocial interventions
- Working with patients' explanatory models
- De-stigmatising
- Focussed on both psychological and social issues
- Aimed to improve depression and anxiety
- Aimed to decrease social isolation

Following our synthesis, we drafted the details of the intervention and developed the training materials for interventions with older people and people from ethnic minority communities.

To ensure our intervention was acceptable and culturally sensitive, we undertook 11 focus groups with a total of 117 service users, members of the community and service providers. South Asian and Somali service user and 3rd sector groups were conducted either through interpreters or by a researcher who spoke Urdu, Hindi and Punjabi. The key findings of the focus groups incorporated into the interventions were: labelling the intervention ‘wellbeing’ rather than ‘mental health’; providing choice of both individual and group interventions; providing home visits for the older person); allowing self-referral; the intervention should be socially orientated, and empower people with skills and tools that they could use in their daily life.

### The wellbeing intervention

The wellbeing intervention focussed on brief cognitive behavioural strategies aimed at decreasing anxiety and depression and social isolation. The title *Wellbeing Intervention* was designed to maximise engagement and reduce stigma. We incorporated a patient-centred interview and shared problem statements, goals and wellbeing plans. The intervention was delivered by *wellbeing facilitators.*

**Figure 2:**
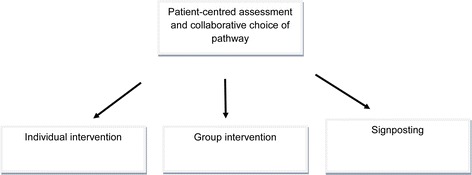
**The wellbeing intervention.**

Participants were offered an initial patient-centred assessment session with a wellbeing facilitator, and collaboratively devised a wellbeing plan. The wellbeing plan specified desired health or social care changes based on self-identified goals. Significant emphasis was placed on the patient as the ‘agent of change’, incorporating patients’ prior experience and coping strategies into the intervention and addressing stigma, expectations, and illness trajectory to better engage patients. Once the goals had been identified, the participant chose up to three ways to obtain support to achieve them: individual sessions with their wellbeing facilitator; group sessions with other participants; or direction (‘signposting’) to appropriate public or voluntary services in their locality (Figure 2).

### Individual intervention

- The individual intervention used cognitive behavioural therapy (CBT), including behavioural activation, cognitive restructuring, applied relaxation and problem solving, with an additional emphasis on decreasing social isolation by encouraging social interaction and activities.
- The delivery mode (face-to-face or telephone) was dependent on participant’s preference.
- Individual interventions comprised up to eight sessions of approximately 30 minutes’ duration over a 16-week period.

### Group intervention

- A wellbeing group, delivered by the wellbeing facilitator and third sector volunteers, focussed on improving mental health through group activities.
- In the groups there was a strong emphasis on improving health and wellbeing, and decreasing anxiety, depression and social isolation. Groups were delivered weekly for one to two hours over an eight to 10 week period.
- The content derived from the development work and focus groups was specifically adapted by the group leaders to be culturally appropriate for the participants. For example older people were offered a Creative Activities group in order to avoid the stigma of mental health terminology [25]. South Asian participants were offered a group session on intergenerational conflicts, such conflicts were mainly focussed on living with in-laws [26,27].

### Signposting

- Signposting to local mental health or social care services (support or drop in centres) or activities which included leisure and educational or employment opportunities. The aim of signposting was to decrease anxiety/depression, reduce social isolation and enhance health and wellbeing through mainstream, socially inclusive activities.
- Participants where only signposting was indicated were seen three times over a 16-week period to assess their success in contacting or attending their preferred activities.

### Role of the wellbeing facilitator

The role of the wellbeing facilitator was to engage, support, advise and actively follow up participants. This included liaison with health and social care professionals who were, or needed to be, involved in the participant’s care, including the participant’s GP. Where the participant was prescribed antidepressant or anxiolytic medication, the wellbeing facilitator would encourage guideline-concordant medication use, in liaison with the GP. Wellbeing facilitators included mental health workers from both the NHS and voluntary sector (NHS included 2 Psychological Wellbeing Practitioners, 2 counsellors, 1 CBT therapist, voluntary sector included 2 health workers both with a counselling qualification).

### Training the wellbeing facilitators

The nine wellbeing facilitators attended a three-day training programme. The training was accompanied by a training handbook which detailed session-by-session content (available from the authors). A significant portion of the training focused on delivering the intervention (individual and group, using fictitious but typical cases of people with anxiety and depression). The research team developed an extensive directory of local services to ensure effective signposting.

### Supervision

All wellbeing facilitators were supervised on a fortnightly basis either face-to-face or by telephone for approximately 30 minutes (depending on location), by clinicians in the trial team.

### Phase 2: Evaluation of the intervention

We evaluated the intervention by carrying out an exploratory randomised controlled trial with a linked qualitative evaluation of views among participants, wellbeing facilitators and supervisors about the acceptability and appropriateness of the intervention.

### Exploratory randomised controlled trial

Our objectives were to estimate key parameters for a definitive trial of the intervention protocol. This included process and rate of recruitment, and intervention delivery, plus preliminary comparisons of outcomes between intervention and usual care participants.

#### Design

Exploratory randomised trial of the effectiveness and acceptability of the wellbeing intervention for common mental health symptoms in (a) elder patients (b) patients from ethnic minority groups, compared to treatment as usual (TAU).

### Method

The exploratory study involved two trials. The core procedures (assessment, allocation, intervention and follow-up) were identical in both trials, but the populations varied. We recruited older adults and ethnic minority participants (South Asians and Somalis) in two Primary Care Trusts in the Northwest of England. Core inclusion criteria for both elders and ethnic minority participants included being registered with one of 16 primary care practices in the two Primary Care Trusts and scoring 10 or more on the PHQ-9 and/or the GAD-7 (threshold for moderate depression and anxiety respectively). Specific inclusion criteria for ethnic minority participants were to be aged 18 or over and to self-identify as being of Somali or South Asian heritage. Elders specific inclusion criteria was for participants to be aged 50 and over. Core Exclusion Criteria for both groups were that participants were not currently deemed to be at significant risk to themselves, or others and did not have significant learning disabilities or cognitive impairment.

### Recruitment

Was through multiple methods, including referrals from GPs, other health professionals, the voluntary sector, or self-referral. Information was made available in relevant languages (Arabic, Bengali, English, Somali, Sylheti and Urdu) and in suitable locations in the community (e.g. surgeries, pharmacies, community centres, libraries, grocery stores and religious centres including churches and mosques). The study was also actively publicised through regular newsletters and open community meetings. Recruitment took place between September 2010 and December 2011.

Details of potential participants were forwarded to the research team, who provided more detailed information about the project and trial, then visited them to discuss the study and confirm consent to participate.

### Ethics

Ethical approval was obtained by the North-West 8 Research Ethics Committee (reference 10/H1003/38).

### Randomisation

Was on a 2 (intervention) to 1 (control) basis, and carried out by an administrator who had no formal connection to the study to ensure concealment of allocation from those assessing eligibility to the study. The recruiting researcher sent details to the administrator, who randomised using random number tables. Separate randomisation schedules were used for ethnic minority and older participants.

### Sample size

We estimated that 50 participants in each trial would be sufficient to assess feasibility, and estimate effect size for future trials.

### Outcome measures

The primary outcome was the CORE-OM [28] a 34-item self report scale designed to measure global distress, including subjective well being, life/social functioning and risk. The CORE-OM has published evidence of reliability and validity [29,30] and has been used in previous UK mental health trials [31].

Secondary outcomes also used scales with published evidence of reliability and validity which have been routinely used in mental health research and routine assessment of outcomes in mental health services [32]. These included measures of depression (Patient Health Questionnaire - PHQ-9) [33]: anxiety (Generalised Anxiety Disorder Assessment - GAD-7) [34]: functioning (Work and Social Adjustment Scale - WSAS) [35], and health related quality of life (EQ-5D) [36] plus a brief demographics form.

These outcome measures have previously been used in non-English speaking populations. Existing translations of these measures were procured for our target languages and in case of non availability we translated and adapted the measure as per published guidelines [37].

### Analysis

Quantitative data were analysed on an intention-to-treat basis. We compared baseline demographic and clinical characteristics of the groups using descriptive statistics, and calculated standardised mean differences to provide an estimate of the treatment effect (mean of intervention group at 20 weeks, minus mean of the control group, divided by the pooled standard deviation). Analyses were conducted in SPSS and Stata.

### Uptake and delivery

To explore uptake and delivery of the intervention we collected data on the interventions that participants chose, treatment uptake and attrition rates.

### Acceptability

To explore acceptability from the patients’ perspectives and to ensure inclusion of patients with a range of baseline characteristics, almost all patients randomised into the trial were invited to participate in interviews. Semi-structured interviews with consenting participants were conducted in patients’ homes by a researcher. A topic guide derived from existing literature and research aims was agreed through discussion within the research team (all researchers with qualitative methodology expertise) which explored key areas including experience of, barriers to and enablers of the wellbeing intervention. This guide provided prompts for use within the interviews, but also allowed for a dialogue to occur within the interview, so allowing the participant's voice to be heard. The researchers conducting the South Asian community interviews spoke both Urdu and Punjabi. Interviews in Bangla and Sylheti were jointly conducted with a Bangladeshi community mental health worker who assisted with the translation. The researchers had participated in training in qualitative methods and were supported and debriefed by experienced qualitative methodologists.

Interviews were audio recorded and lasted between 45 and 60 minutes. In addition we conducted interviews with wellbeing facilitators and supervisors. An interview schedule developed by the trial team was used and all interviews were audio recorded.

## Results

### Recruitment

The trial failed to meet the recruitment target of 100. Only 57 eligible participants were recruited and only 1 individual of Somali heritage was recruited. We continue to use the term ‘ethnic minority’ to describe this group, although the vast majority were South Asian.

**Figure 3:**
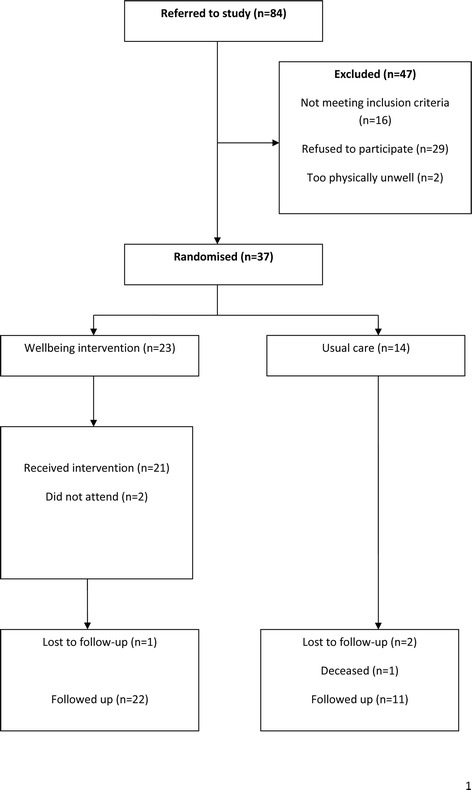
**CONSORT flowchart for the elders’ sample.**

In the elders group, 84 patients were referred (Figure 3). Fifty two (62%) referrals were by GPs, 11(13%) were self-referrals 7(8%) by primary care mental health teams, 4(5%) by voluntary organisations; and 10 (12%) by others (including community matrons, housing association staff and practice nurses). Thirty seven (44%) were eligible and randomized to be allocated to either control or intervention. At 20 weeks 33 (89%) completed follow-up (96% in the intervention and 71% in the control).

**Figure 4:**
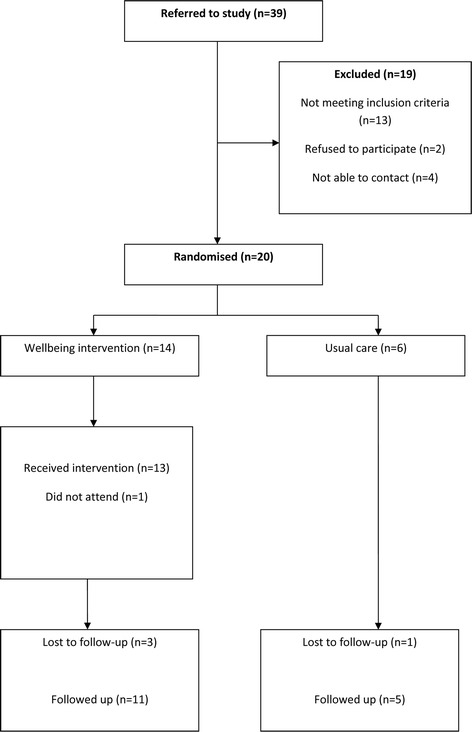
**CONSORT flowchart for the Ethnic minority sample.**

In the ethnic minorities group, 39 patients were referred (Figure 4). Of these 20 (51%) were referred by GPs, 8 (20%) were self-referrals 2 (5%) by primary care mental health teams, 5 (12%) by voluntary organisations and 4 (10%) by others. Twenty (51%) were randomised. At 20 weeks, 16 (80 per cent) completed follow-up (79% in the intervention group and 83% in the control).

Baseline socio-demographic characteristics of the elders sample are shown in Table 1. Most respondents were female, with a mean age of 64, nearly three-quarters were retired or otherwise not working, and most were not involved in community activities. Levels of distress were relatively high, with a mean CORE score of around 20 and a mean PHQ 9 score of 18.

**Table 1:** **Socio-demographic characteristics of patients included in the AMP feasibility study: elders sample**

		**Wellbeing intervention n = 23**	**Usual care n = 14**	**Total n = 37**
**Sex**	Male	6 (26.1%)	3 (21.4%)	9 (24.3%)
	Female	17 (73.9%)	11 (78.6%)	28 (75.7%)
**Age mean (sd) range**		65.64 (10.29) 50-84	60.70 (8.16) 53-78	63.77 (9.73) 50-84
**Ethnicity**	White British	20 (87.0%)	13 (92.9%)	33 (89.2%)
	White other	2 (8.7%)	1 (7.1%)	3 (8.1%)
	Mixed	1 (4.3%)		1 (2.7%)
**Engaged in community activities**	Yes	3 (13.0%)	6 (42.9%)	9 (24.3%)
	No	20 (87.0%)	8 (57.1%)	28 (75.7%)
**Baseline measures** ^**1**^ **mean (sd)**	PHQ9^2^	17.70 (5.14)	19.00 (5.35)	18.19 (5.19)
	GAD7	15.91 (4.31)	13.20 (6.12)	14.88 (5.16)
	WSAS	26.00 (9.48)	22.86 (13.35)	24.81 (11.03)
	EQ5D	0.29 (0.36)	0.31 (0.45)	0.30 (0.39)
	EQ5D health state (0–100)	44.65 (19.59)	53.93 (25.81)	48.16 (22.27)
	CORE-OM (mean × 10)^3^	19.85 (6.72)	20.00 (6.90)	19.91 (6.69)

^1^All health scales are scored so that a high score is indicative of poor health, apart from EQ5D.

^2^PHQ9 scores of 10+ are usually indicative of clinically significant depression, and scores of >20 more severe major depression.

^3^CORE-OM scores of 25 are usually considered indicative of severe distress.

Statistical significance testing of baseline differences is not recommended in trials generally and is inappropriate with small numbers when large differences between groups are likely to be not statistically significant because of limited power. Visual inspection of the data indicates reasonable comparability in terms of gender, age and ethnicity, but differences in employment and community engagement. Importantly, baseline levels of distress on the CORE (primary outcome) and depression were comparable, although there were differences in other health measures.

Baseline socio-demographic characteristics of the ethnic minority sample are shown in Table 2. All participants were female, with a mean age of 40, with most from Pakistani or Bangladeshi groups. Twenty per cent had a degree, and nearly one third were involved in community activities. Levels of distress were relatively high, with a mean CORE score of 25 and mean PHQ score of 19.

**Table 2:** **Socio-demographic characteristics of patients included in the AMP feasibility study: BME sample**

		**Wellbeing intervention n = 14**	**Usual care n = 6**	**Total n = 20**
**Sex**	Male	0 (0%)	0 (0%)	0 (0%)
	Female	14 (100%)	6 (100%)	20 (100%)
**Age mean (sd) range**		38.92 (9.29) 25-56	43.02 (14.50) 21-58	40.15 (10.87) 21-58
**Ethnicity**	Pakistani	6 (42.9%)	4 (66.7%)	10 (50%)
	Bangladeshi	6 (42.9%)	2 (33.3%)	8 (40%)
	Punjabi Indian	1 (7.1%)	0 (0%)	1 (5%)
	Somali	1 (7.1%)	0 (0%)	1 (5%)
**Engaged in community activities**	Yes	3 (21.4%)	3 (50%)	6 (30%)
	No	11 (78.6%)	3 (50%)	14 (70%)
**Baseline measures mean (sd)**	PHQ9	19.36 (3.20)	19.50 (1.76)	19.40 (2.80)
	GAD7	18.07 (3.52)	17.83 (1.17)	18.00 (2.97)
	WSAS	28.85 (8.11)	26.83 (8.35)	28.21 (8.01)
	EQ5D	0.21 (0.36)	0.20 (0.49)	0.20 (0.39)
	EQ5D Health state (0–100)	17.14 (12.51)	17.50 (11.29)	17.25 (11.86)
	CORE-OM	25.01 (5.86)	24.92 (4.56)	24.98 (5.38)
	(mean × 10)			

Visual inspection of the elder and ethnic minority sample data indicates reasonable comparability in terms of age and education, but differences in ethnicity, and social engagement (although these are very sensitive to the small numbers in the groups). Importantly, baseline levels of distress on the CORE (primary outcome), depression and the other health measures were comparable. It is notable that the PHQ9 scores in the South Asian and elder samples are comparable, but the CORE scores were much higher in the South Asian sample.

### Uptake and delivery of the intervention

Of the 37 participants allocated to the wellbeing intervention, three did not attend any session: one participant moved and two were not contactable despite numerous attempts by the facilitators.

Despite being offered sessions face-to-face or by telephone, all participants opted for face-to-face sessions. Of the 34 participants who received an intervention the pathways selected were: 15 individual only; 12 individual and signposting; 5 group, individual and signposting; 1 group and signposting; and 1 signposting only. The mean number of sessions attended was 6.3 (range 1–19). The mean total time of sessions was 326 minutes (range 60–790 minutes).

### Outcomes

#### Elders

The 20 week outcome data for the elders is shown in Table 3. Caution must be exercised in interpretation of outcome data in a feasibility study, as the small numbers mean that baseline differences can occur through chance, and the precision of the estimates is necessarily limited.

**Table 3:** **Baseline and 20 week outcome data for elders’ sample**

	**20 weeks**	**20 weeks**
Outcome	Wellbeing intervention	Usual care
	(M, SD, n)	(M, SD, n)
CORE-OM	14.32, 8.37, 22	19.71, 8.58, 11
GAD7	11.41, 6.96, 22	12.27, 8.17, 11
PHQ9	11.82, 8.05, 22	16.55, 6.25, 11
WSAS	18.20, 11.84, 22	23.55, 14.30, 11
EQ5D	0.40, 0.44, 22	0.27, 0.44, 11

**Figure 5:**
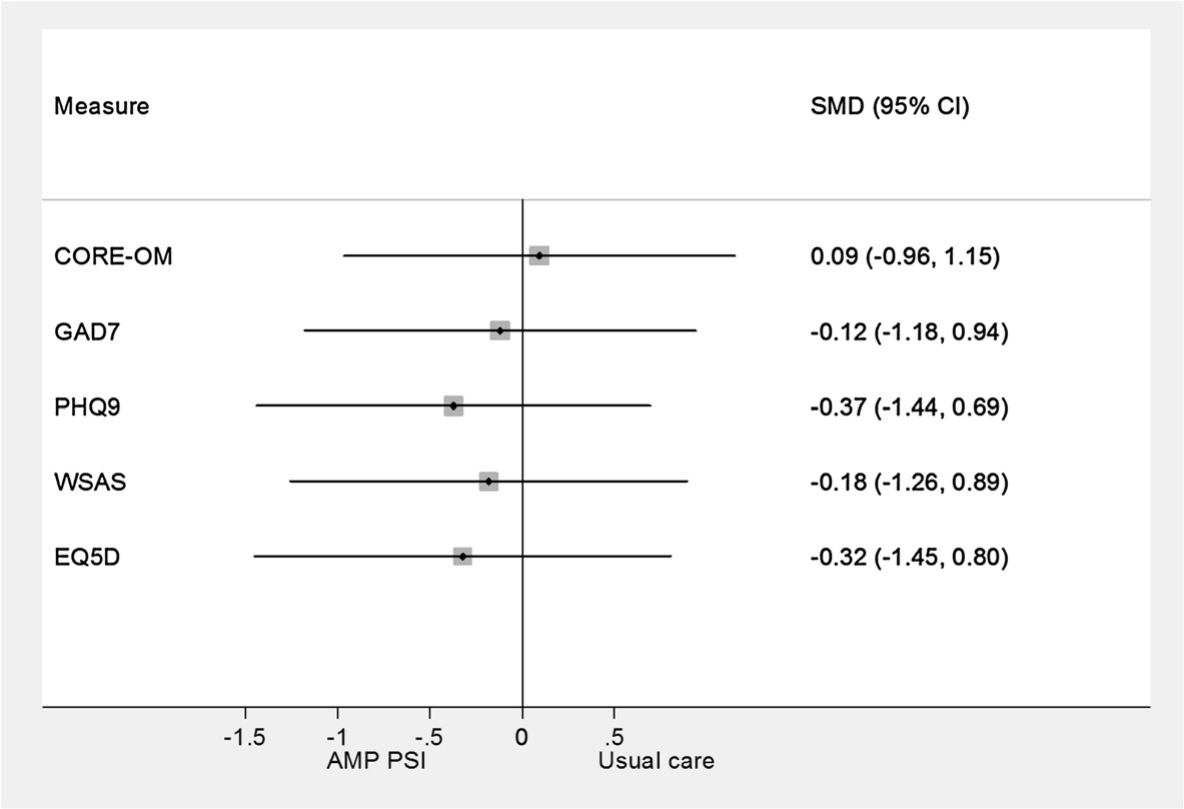
**Forest plot of effects of well being intervention on elders’ sample.**

The results suggest that the group receiving the wellbeing intervention improved compared to the group receiving usual care. The results are plotted in Figure 5 using a standardised mean difference (effect size) measure. All results are in the direction of greater benefit in the intervention group. The largest effects were found on psychological distress (CORE-OM) and depression (PHQ9), with smaller effects on functioning (WSAS), health related quality of life (EQ5D) and minimal impacts on anxiety (GAD-7). The small numbers mean that these results are not statistically significant but the magnitude is fairly substantial compared to other psychosocial interventions in primary care [28]. These results are not adjusted for any potential baseline differences.

### Ethnic minority groups

The 20 week outcome data for the sample is shown in Table 4. Again, caution must be exercised in interpretation of outcome data in a feasibility study, as the small numbers mean that baseline differences can occur through chance, and the precision of the estimates is necessarily limited.

**Table 4:** **Baseline and twenty week outcome data for the Ethnic Minority sample**

**Outcome**	**Wellbeing intervention**	**Usual care**
	**(M, SD, n)**	**(M, SD, n)**
CORE-OM	20.98, 7.40, 11	20.32, 6.32, 5
GAD7	13.45, 4.53, 11	14.00, 4.64, 5
PHQ9	13.99, 4.91, 11	16.00, 6.44, 5
WSAS	22.30, 11.93, 10	24.40, 10.45, 5
EQ5D	0.35, 0.46, 8	0.21, 0.38, 5

**Figure 6:**
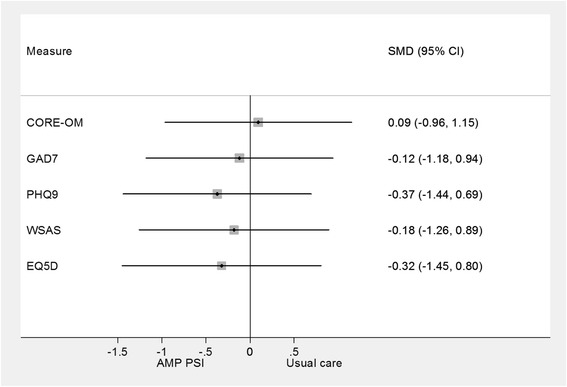
**Forest plot of effects of well being intervention on the Ethnic minority sample.**

The results are plotted in Figure 6 using a standardised mean difference (effect size) measure. The effects are generally smaller than in the elders’ sample. The largest effects are on depression (PHQ9) health related quality of life (EQ5D) and functioning (WSAS), where the impacts were similar to comparative data from primary care [28]. The effects on psychological distress (CORE-OM) and anxiety (GAD-7) were small and unlikely to be of clinical significance. These results are not adjusted for any potential baseline differences.

### Acceptability of the intervention

We carried out 39 interviews with trial participants (Table 5), 5 of the 7 wellbeing facilitators and all 3 supervisors. Interviews were transcribed verbatim, and analysed using a Framework Approach [38] by familiarisation, indexing, charting and interpreting the data as a team. Our thematic framework focuses on acceptability and appropriateness of the intervention. Direct quotations have respondent identifiers, with characteristics removed to ensure anonymity.

**Table 5:** **Characteristics of interviewed trial participants**

**Group**	**Location**	**Gender**	**Ethnicity**	**Age range**
*Elders*	Croxteth	7 female	7 White British,	57-79
		1 male	1 White German	
*Elders*	Wythenshawe	7 male	13 White British	51-85
		9 female	1 White British/ Polish	
			1 Australian	
			1 Indian/Caribbean/English	
*South Asian*	Longsight	14 female	7 Bangladeshi,	24-60
			6 Pakistani,	
			1 Indian	
*Somali*	Picton	1 female	Somali	-

### Accessing the intervention

#### Deciding to participate

Recommendation from the GP was important in deciding to participate,

*“if your doctor says it’s OK” C04 (Elder) and particularly in the case of South Asian women, discussion with family members.*

*“I’ve spoken to my husband about it. He said if that is what your doctor has recommended then go and try it. If you speak about your problems may be your mind will be ‘refreshed’ and you will feel better.”* L005 (Bangladeshi)

*However self-referral also proved to be an important mode of access, even though some people may need continuous informational and motivational support to really gain access to help by self-referring. Of note is that previous studies found that self-referral and more particularly non-stigmatising self-referral, increased access for ethnic minority communities* [39,40]*.*

“*they never recommended it to us in the first place you know cause they were in one of the practices in included in the programme but they never recommended it to us uh you know so that’s why I thought well I’d go and see if we were eligible to go on it.*” W019 (Elder)

### Engaging participants

The wellbeing facilitator’s were encouraged to contact people on a number of occasions to work at engaging them, as described in other models of collaborative care:

*“we were more proactive in contacting people than we would have been usually within the service so I think they might have been referred to the service but I doubt they would have actually been seen for assessment or would have continued with treatment anyway.”* Wellbeing facilitator 01 (Elders)

### Locating therapy

For older people in particular, travelling to a venue was sometimes challenging and required some planning:

*“I wouldn’t be able to get the bus.... I can’t let Anon take me cause he’s got appointments with both children cause they’re um asthmatic.”* W001 (Elder)

For both elders, and South Asian women, the flexibility of the wellbeing facilitators in being able to see people at home was important:

*“I said I can’t go anywhere because I have trouble walking. I said I would prefer for someone to come and visit me in my house, so then that’s what they did.”* L008 (Bangladeshi)

*“I think we also picked up a lot more people who were housebound or with physical illnesses.”* Supervisor 1 (Elders)

*“In my Mental Health Worker role I would only be able to see people in a health centre. If they required a home visit then that would be something that to be honest, our team wouldn’t usually do. And can be a massive barrier for people. So that was good to be able to see people in a venue that they preferred; whether that was at their GP surgery or at home.”* Wellbeing facilitator 02.

Both elders and South Asian women preferred face-to-face to telephone contact, and almost all the therapy in both groups was provided in this way.

*“No no I don’t like talking on the phone****Int****: “Ah ok can you say more about that… “”I like to see people’s faces when I am talking… Because they could be saying one thing and meaning another.”* C034 (Elder)

The atmosphere of the venue for therapy (both one to one and group) provided by a third sector organisation was very much appreciated:

*“A warm friendly feeling....you go some places you don’t get that warm friendly feeling at all it is a friendly atmosphere there’s I’ve not found anybody stabbing one another’s back.”* W004 (Elder)

### Promoting access

The South Asian participants had some important suggestions about how they thought access to the intervention could have been better promoted in their community.

*“I think you need to advertise it in a way so that other people in the family do not have a problem with this type of programme. I think you can also get family support by using Islam to promote the issue/ programme.”* L027 (Bangladeshi)

*“Most of the people are not literate so audio information should be available.”* L002 (Pakistani)

*“I think you have to involve the husbands too and educate them about what you are doing and why.”* L003 (Bangladeshi)

### Appropriateness of therapy

#### Cultural fit

This was a major issue for the South Asian women. Women who spoke Urdu, Punjabi or Hindi could be seen by a wellbeing facilitator who spoke their language, while Bangla, Sylheti and Somali speakers were seen with an interpreter. Some participants preferred their well being practitioner of their own ethnicity.

*“I would prefer someone from our own ethnicity and someone who knows the culture well. There are so many sensitive issues that they won’t be able to understand and plus the way we talk in our own language you can’t say things fluently if you don’t know the language well*.” L006 (Pakistani)

However others felt that empathy and understanding were more important than the ethnicity of the well being facilitator:

*“I think she is very supportive she is a very nice girl. Even though she is a White person I know that she sympathises with me and understands my illness. When i am uncomfortable you can tell that she feels my pain, she does not have to say it but you can just tell can’t you?*” L008 (Bangladeshi)

And personal choice was important:

*“Sometimes I suppose yes it matters as they do understand your culture and your cultural values and if language is a barrier then its best to talk with someone who understands and speaks the same language, but …people may prefer someone outside their community. I think it’s a matter of personal choice.”* L009 (Pakistani)

The therapy team were also concerned by the lack of therapy services available for speakers of Bengali, Sylheti and Somali.

*“One of the Bangladeshi women really needed to be referred* [on] *for counselling …And we couldn’t even find a counsellor who could speak her language.”* Supervisor 2 (South Asians)

Although most experiences were positive, this was not universal and there were comments from elders about whether a young wellbeing facilitator would really able to empathise with their problems:

*“I think she was trying the best she could but I did find her overpowering […] I just said I found it she was asking me to do things that I didn’t really feel capable of doing....*“*I didn’t feel able to talk to her because I felt she was too young and she wouldn’t understand.*” W014 (Elder)

### Content of the intervention

Generally, across both older people and the South Asian women who participated in the intervention, the specific content of the psychological therapy was appropriate acceptable and perceived to be helpful.

*“*[Wellbeing facilitator] *does understand and she does not ask anything inappropriate. I have been going for the sessions and things are not against our culture or religion.”* L023 (Bangladeshi)

It was possible to identify, from the interviews, the active elements of behavioural activation being used and sustained:

“I think I do set myself little goals you know in the week

Interviewer: “*Hmm and do you think you’ll be able to sustain that?”*

*“I’m hoping to I mean [facilitator] said I would be you know I should be able to and you know and I have and she said you know she saw a marked improvement in me and I have grown in confidence as well.”* W019 (Elder)

*“She sort of got me going doing things..... balancing me day out and making a chart out of the week and doing that when I feel a bit down now I refer back to that and make a chart for myself just to get me moving.”* W004 (Elder)

The need for also signposting to local agencies was also however apparent:

*“if there are people who could help us sort out our other domestic and financial issues as well nothing better could be done.*” L006 (Pakistani)

### Group intervention

Groups were established in partnership with third sector organisations and were popular with those who attended:

*“I learnt new things and made friends as well. I had a good time and learnt to manage stress.”* L011 (Pakistani)

In one area a single group ran, which utilized a combination of active engagement in crafts, and therapeutic intervention to move participants forwards. It was co-facilitated by an wellbeing facilitator and a worker from our partner third sector organisation. This type of group, run at an Age Concern base (an older persons NGO), was developed to overcome the perceived stigma associated even with a ‘wellbeing’ group.

Interviewer: “*so when you had the group, the group where you were making things and there was* [Age Concern group leader] *and* [wellbeing facilitator] *leading it, did you feel that you were being offered support?”*

*“Yes”*

Interviewer: *“Through* [wellbeing facilitator]?”

*“Through* [wellbeing facilitator].”

Interviewer: “*Right how did that support work?”*

*“Well she chatted to you and gave you encouragement….And asked how you felt the day if you felt a bit down she’d talk through things with you.”* W004 (Elder)

However not all people felt able to engage in a group:

*“I would like to try to attend a group session but honestly I don’t feel like talking about my mental health issues in front of other people. They gossip a lot and that is why I don’t mix up with people.*” L014 (Pakistani)

*“I would feel vulnerable.”* W034 (Elder)

### After the intervention

There was also a sense that for all participants, the intervention (group and individual) was not long enough,

*“I’m sorry but when you’re suffering with mental health like myself you’ve suffered with it for years, six weeks isn’t going to cure it.*” W005 (Elder)

“…*a fixed amount of sessions*…*Isn*’*t always the best way to go* ‘*cos some people might need a few more*, *some people might need less.*” W027 (Elder)

However, some participants became actively involved in other activities and expressed desires to volunteer with these groups.

“*I started the craft group and um been doing sort of things since.*” W005 (Elder)

“*I was you know really well impressed*, *they*’*ve asked me to be a volunteer*.” W19 (Elder)

“*I am offering you that I can work as a volunteer I know what depression is what people go through and what can help to recover*.” L02 (Pakistani)

The wellbeing facilitator’s also noted that other types of intervention might be helpful for people who could not engage in groups, for longer term support:

“*I suppose one thing that kind of came out of it for me was*… *for people who maybe don*’*t want to become involved with a group for whatever reason that there is a need for kind of an informal support like a befriending service*.” *W*ellbeing facilitator 4 (Elders)

## Discussion

Our plan to develop a new intervention in close collaboration with the target communities was successful. The intervention was generally acceptable to patients, and there was evidence of adherence among those who attended. The majority of patients preferred one-to-one sessions, though the options for group work and signposting were valued.

Evidence from our preliminary analyses of outcomes indicated that patients offered the wellbeing intervention showed greater improvement than those offered usual care. In both groups, our measure of depression outcomes (PHQ9) demonstrated change of clinically significant magnitude. The magnitude of difference was comparable to other psychosocial interventions in primary care [28] and will be helpful in estimating sample size for a future definitive trial. It is noteworthy that neither group demonstrated large benefits in anxiety symptoms, despite the intervention being designed to improve both anxiety and depression. There was preliminary evidence that the intervention impacted on wider function and quality of life in both groups, although the results must be viewed with caution due to the limited sample size. Although similarities and differences in outcomes between the two groups also exist, interpretation of these patterns should also be cautious, as the measures have generally been not been developed for, or extensively validated with ethnic minority populations. There is a danger that such scales fail to assess the constructs used in different groups around mental health and distress.

The trial tested a ‘complex intervention’ which was designed to reflect the multiple challenges faced by these groups (including chronicity of problems, social isolation, stigma, and different explanatory models), and to try and balance the expressed needs of the client group, and the skills and knowledge of the research team. There is an argument that achieving all this in the context of a time limited primary care intervention is over ambitious, and that greater focus would be preferable. An additional issue with the evaluation of multicomponent ‘complex interventions’ is that the core mechanisms of effect are difficult to identify. The qualitative work reported here is an attempt to highlight patient experience as a guide to what aspects are most and least effective, but accurate identification remains a significant methodological challenge.

Despite considerable effort on the part of the research team, our recruitment rates were lower than anticipated. Recruitment to primary care trials in the United Kingdom is routinely problematic and particularly difficult in trials of mental health [41-43]. Therefore, determining recruitment rates and the most effective recruitment strategies are critical steps in the development of a definitive trial. However, available evidence to support recruitment strategies is sparse [44].

In other mental health trials we have found that successful recruitment has been achieved by mass screening lists of registered primary care patients [45], which results in large numbers of recruits, although the overall response rate tends to be low. While it might be possible to adopt this strategy for older people, who do tend to register in primary care, our knowledge of the barriers to recruitment identified in earlier stages of the AMP project made such an approach problematic. Mass screening would also be less likely to reach under-served members of ethnic minority communities, who may not have registered as patients with primary care teams or if they have, may not be readily identifiable from practice lists. Therefore, the current recruitment rates should be seen in a context where one of the most effective strategies was not available.

Some groups were particularly affected by low recruitment. Although South Asian males were referred none were randomised, although it is not clear whether this reflects attitudes to the intervention, or to the research. The trial in the Somali locality recruited only one eligible patient. Low recruitment in this group may have reflected the overall study design (outside the trial reported here), since the community engagement interventions were not active here [15]. That community engagement would be more important in ethnic minority recruitment, and less so in older peoples recruitment, is anticipated by the literature [44,46,47], We address these issues in more detail elsewhere [7]. The additional complexities of pre and post migration stressors experienced by the Somali community as a less established community, seeking asylum in the UK arguably add to the difficulties of recruitment and of designing acceptable and accessible interventions [48-50]. This correspondingly limits any potential transferability of our current findings to wider ethnic minority groups.

There is an argument that the recruitment problems in part reflected the decision to develop an intervention for two different groups. Despite the potential advantages in terms of cost and wider implementation, this may have lessened the acceptability of the intervention in each constituent group.

Cultural issues were important. On the one hand, it is important to ensure that wellbeing facilitators are seen as culturally appropriate by the local communities: this applies as much to older indigenous populations as to particular ethnic minority groups. On the other hand, our experiences may support an argument for reducing the cultural specificity of wellbeing interventions. There is a trade-off between specificity and potential sample size (most evident for the Somali community). Reducing specificity would also limit the risk of a particular community seeing itself as stigmatised, rather than prioritised, by the offer of an intervention. However it is also clear that for the South Asian participants cultural specificity was valued and seen as important. Different strategies may therefore be needed to engage particular communities.

It should be noted that attrition rates at the first follow up were not unusually high, despite the difficulties engaging these groups and others living in deprived localities [51]. Minimising attrition is essential to ensuring that baseline comparability provided through randomisation is maintained, and that sample size achieved at recruitment is not significantly reduced in the main analysis. The study suggests that although there is still work to be done in encouraging these groups into trials, their experience once recruited does not lead to particular problems of retention.

## Conclusions

The intervention does show preliminary evidence of effectiveness in people who do engage, although clearly major problems remain in terms of encouraging higher rates of recruitment to ensure those benefits have sufficient reach into population in need. The alternative of developing specialist teams to deliver targeted interventions may provide greater benefits in areas with sufficient need [52]. However, developing our understandings of how existing local services can better tailor mental health treatment is a necessary first step in developing services that are responsive to the needs of our increasingly diverse communities.

## Abbreviations

CBT: Cognitive-behavioural therapy
TAU: Treatment as usual
CORE-OM: Clinical Outcomes in Routine Evaluation
PHQ-9: Patient Health Questionnaire
NHS: National Health Service
GAD-7: Generalised Anxiety Disorder Assessment – 7-item scale
EQ-5D: European Quality of Life – 5 Dimensions
GP: General practitioner
WSAS: Work and Social Adjustment Scale
